# Monocyte-Platelet Interaction Induces a Pro-Inflammatory Phenotype in Circulating Monocytes

**DOI:** 10.1371/journal.pone.0025595

**Published:** 2011-10-12

**Authors:** Gabriella Passacquale, Padman Vamadevan, Luis Pereira, Colleen Hamid, Valerie Corrigall, Albert Ferro

**Affiliations:** 1 Cardiovascular Division, Department of Clinical Pharmacology, King's College London, London, United Kingdom; 2 Academic Department of Rheumatology, King's College London, London, United Kingdom; King's College London, United Kingdom

## Abstract

**Background:**

Activated platelets exert a pro-inflammatory action that can be largely ascribed to their ability to interact with leukocytes and modulate their activity. We hypothesized that platelet activation and consequent formation of monocyte-platelet aggregates (MPA) induces a pro-inflammatory phenotype in circulating monocytes.

**Methodology/Principal Findings:**

CD62P^+^ platelets and MPA were measured, and monocytes characterized, by whole blood flow cytometry in healthy subjects, before and two days after receiving influenza immunization. Three monocytic subsets were identified: CD14^+^CD16^−^, CD14^high^CD16^+^and CD14^low^CD16^+^. The increase in high sensitivity C-reactive protein post-immunization was accompanied by increased platelet activation and MPA formation (25.02±12.57 vs 41.48±16.81; p = 0.01), along with enhancement of circulating CD14^high^CD16^+^ cells (4.7±3.6 vs 10.4±4.8; p = 0.003), their percentage being linearly related to levels of CD62P^+^-platelets (r^2^ = 0.4347; p = 0.0008). In separate *in vitro* experiments, co-incubation of CD14^+^CD16^−^ cells, isolated from healthy donor subjects, with autologous platelets gave rise to up-regulation of CD16 on monocytes as compared with those maintained in medium alone (% change in CD14^+^CD16^+^ cells following 48 h co-incubation of monocytes with platelets was +106±51% vs monocytes in medium alone; p<0.001). This effect correlated directly with degree of MPA formation (r^2^ = 0.7731; p<0.0001) and was associated with increased monocyte adhesion to endothelial cells. P-selectin glycoprotein ligand-1 (PSGL-1) blocking antibody, which abrogates MPA formation, abolished these effects, as did the cyclooxygenase (COX)-2 selective inhibitor NS-398, aspirin and the EP1/EP2-selective antagonist AH6809.

**Conclusions/Significance:**

These data suggest that MPA formation, as occurs in the blood under pro-inflammatory conditions, expands the pool of circulating CD14^high^CD16^+^ monocytes in a COX-2 dependent manner, and these monocytes exhibit increased adhesion to endothelium. Our findings delineate a novel mechanism underlying the pro-inflammatory effect of platelet activation.

## Introduction

Monocyte-platelet aggregates (MPA) are heterotypic complexes detectable in the peripheral blood which form in response to platelet activation [1]. Accordingly, circulating MPA level increases in patients with acute thrombotic events, such as myocardial infarction [1], [2] or stroke [3], [4], as well as in subjects with underlying atherothrombotic risk factors including hypertension [5] and diabetes [6]. Circulating MPA are also increased in patients with a variety of autoimmune disorders [7]. The level of MPA reflects the degree of platelet hyperactivity thus providing a robust index of blood thrombogenicity [2]. However, cross-talk between platelets and monocytes is now regarded as a crucial pathophysiological mechanism linking thrombosis and inflammation and is believed to mediate, at least in part, the pro-inflammatory action of activated platelets. Indeed, *in vitro* studies have shown that contact with platelets enhances cytokine and prostanoid production by monocytes [8]–[10], as well as their adhesiveness to the vascular endothelium [11]. However, the importance of monocyte-platelet interaction in human inflammatory pathophysiology, as well as the precise mechanisms by which such interaction modulates monocytic function, remain unclear.

Circulating monocytes comprise different sub-populations with distinct infiltrative and migratory properties, that can be distinguished on the basis of differential expression of the surface markers CD14 and CD16 [12], [13]. We hypothesized that a mild systemic inflammatory stimulus will increase circulating MPA, thus inducing a pro-inflammatory change in monocyte phenotype.

The aims of this study were therefore firstly to investigate in vivo the effect of a mild acute inflammatory stimulus, namely influenza immunization [14], on circulating MPA level and monocyte phenotype; and secondly to determine in vitro the underlying mechanism by which monocyte-platelet interaction modulates monocyte phenotype and function.

## Results

### Influenza immunization causes an increase in circulating MPA and a shift in circulating monocytes towards CD16 positivity

**Figure 1:**
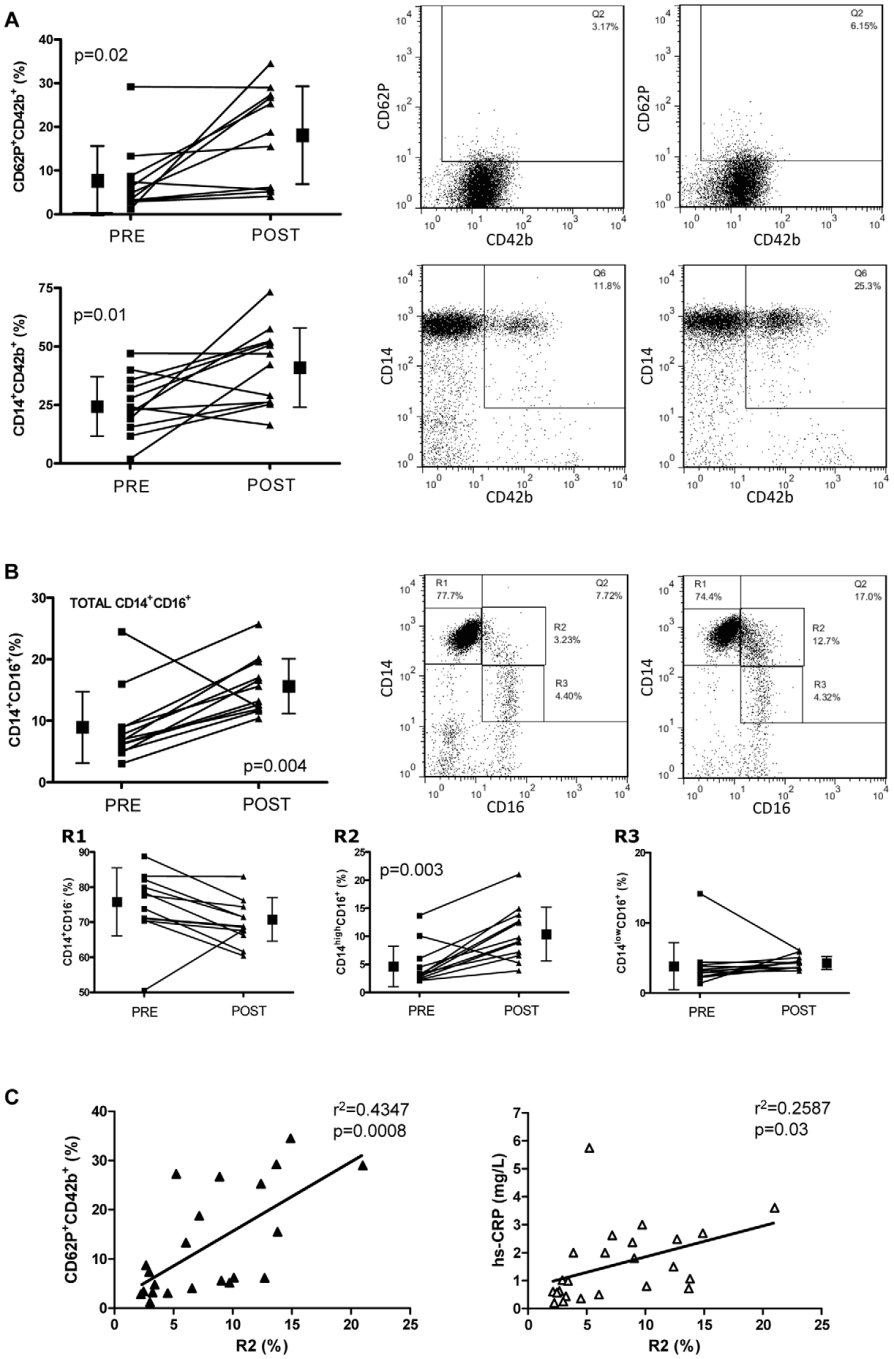
Effect of influenza immunisation on platelet activation and monocyte phenotype. Percentage of CD62P^+^platelets and MPA (**A**), and monocyte characterization (**B**), measured at baseline (PRE) and 2 days after influenza immunization (POST). Figures show representative dot plots obtained from flow cytometry, as well as accumulated data from n = 12 experiments. Monocytic subsets are designated R1 (CD14^+^CD16^−^), R2 (CD14^high^CD16^+^) and R3 (CD14^low^CD16^+^). (**C**), Regression analyses for CD14^high^CD16^+^ monocytes (R2) *vs* CD62P^+^platelets and *vs* hs-CRP.

Administration of the influenza vaccine induced an increase in hs-CRP, as expected (0.57±0.26 mg/L at baseline vs 2.94±1.44 mg/L two days post-immunization, p = 0.002). In line with this, we found an increase in degree of platelet activation, as reflected by CD62P^+^ platelet positivity and level of MPA (Figure 1A). We also observed a change in distribution pattern of monocyte subsets, with an expansion of the pool of CD14^+^CD16^+^ monocytes (Figure 1B). Within this double positive pool, the subset of CD14^high^CD16^+^ monocytes showed the greatest increase in percentage (from 4.7±3.61% to 10.44±4.79%, p = 0.003), whilst the CD14^low^CD16^+^ subset did not change significantly (Figure 1B).

In examining the relationship between the percentage of CD14^high^CD16^+^ monocytes and other variables examined (hs-CRP, CD62P^+^platelet and MPA levels), a linear correlation was found between the CD14^high^CD16^+^ subpopulation and both hs-CRP and percentage of CD62P^+^ platelets. However, levels of CD14^high^CD16^+^correlated more closely to percentage of CD62P-expressing platelets than with hs-CRP concentration (Figure 1C).

### Detailed characterization of circulating monocytic subsets and MPA

**Figure 2:**
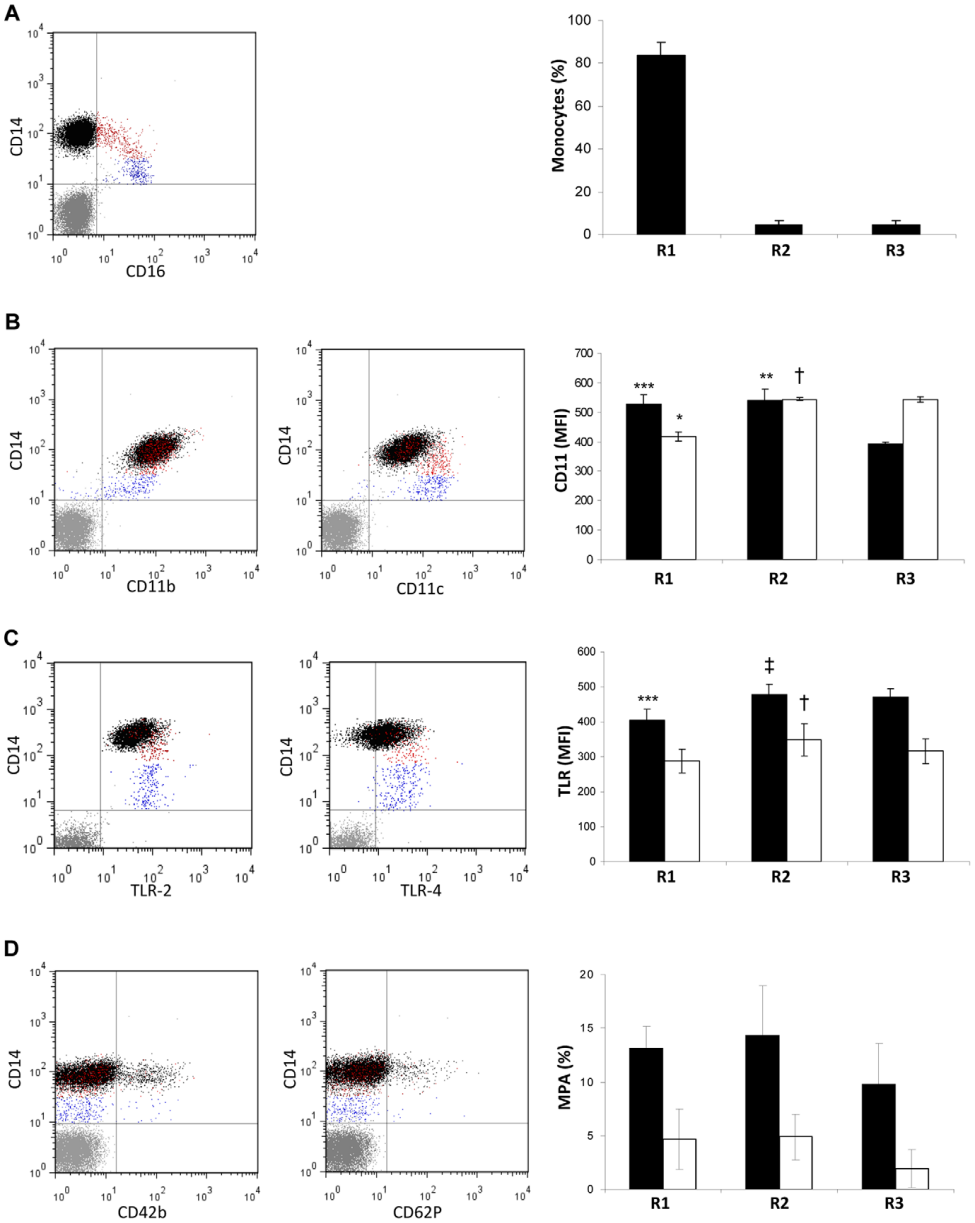
Circulating MPA and monocyte characterization. (**A**) Percentage of the different circulating monocyte subpopulations, with a representative whole blood flow cytometry dot plot showing CD14 and CD16 expression, as well as accumulated data from n = 10 experiments presented graphically. (**B**) Representative flow cytometry dot plots for CD14 and CD11b (left) or CD11c (right) in the different monocyte subsets. Graph displays MFI values for CD11b (filled bars) and CD11c (open bars) in each subset (n = 10). (**C**), Representative flow cytometry dot plots for CD14 and TLR-2 (left) or TLR-4 (right) in the different monocyte subsets. Graph displays MFI values for TLR-2 (filled bars) and TLR-4 (open bars) in each subset (n = 10). (**D**) Representative flow cytometry dot plots for CD14 and CD42b (left panel) or CD62P (right panel). Graph shows data accumulated from n = 10 experiments illustrating the % MPA formed by the different monocytic subsets, as determined from CD14^+^CD42b^+^ (filled bars) or CD14^+^CD62P^+^ events (open bars). In black, red and blue are CD14^+^CD16^−^ (R1), CD14^high^CD16^+^ (R2) and CD14^low^CD16^+^ (R3). *,**, *** p<0.05, <0.01 and <0.001 respectively *vs* R3. ^†, ‡^ p<0.05 and <0.01 respectively *vs* R1.

As shown above, on the basis of CD14 and CD16 expression, circulating monocytes were distinguished into CD14^+^CD16^−^, which constituted the majority, with smaller contributions from CD14^+^CD16^+^ cells, comprising CD14^high^CD16^+^ and CD14^low^CD16^+^ subtypes (Figure 2A). All of these monocytic subsets expressed the adhesion molecules CD11b and CD11c on their surface. However, the level of expression of CD11b was higher on CD14^+^CD16^−^ cells than on CD14^low^CD16^+^ monocytes, whereas these latter cells expressed an increased level of CD11c compared to the CD14^+^CD16^−^ subpopulation; the CD14^high^CD16^+^ cells demonstrated an intermediate phenotype, expressing both CD11b and CD11c at high level (Figure 2B). CD14^high^CD16^+^ cells also represented the monocytic subtype with the highest expression level for both TLR-2 and TLR-4. Specifically, all the different subsets of monocytes were positive for TLR-2, but the MFI value was higher in CD14^high^CD16^+^ cells than in the other monocyte subtypes. TLR-4 was found to be expressed by 76.35±12.3% of CD14^+^CD16^−^ monocytes, 91.7±5.8% of CD14^high^CD16^+^ (p = 0.02 vs CD14^+^CD16^−^) and 82.03±7.26% of CD14^low^CD16^+^ cells (p>0.05 vs CD14^+^CD16^−^). Moreover, CD14^high^CD16^+^ cells exhibited higher MFI for TLR-4 compared to the other monocytic subtypes (Figure 2C).

Circulating MPA, as defined by double positivity for CD14 and CD42b, accounted for 15.2±6.7% of the monocytic population. No significant difference was observed in the ability of the different monocyte subpopulations to interact with platelets (Figure 2D) although, as expected, the number of CD14^+^CD62P^+^ events was less than the number of CD14^+^CD42b^+^ events.

### Monocyte-platelet interaction leads to a phenotypic change of CD14^+^CD16^−^ monocytes towards CD14^+^CD16^+^

**Figure 3:**
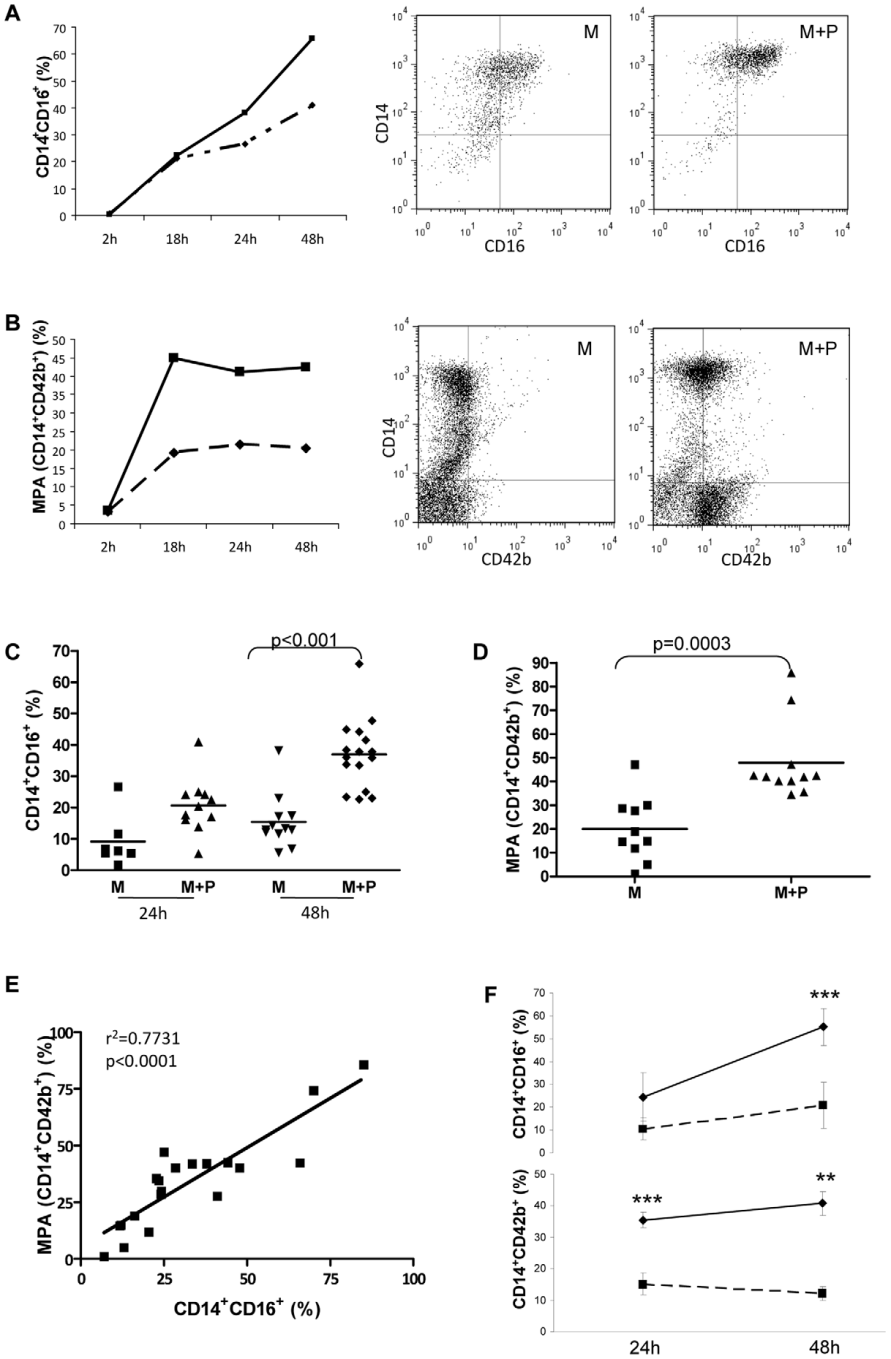
Effect of platelets on monocyte phenotype. Percentage of CD14^+^CD16^+^ monocytes (**A**) and MPA (**B**), following incubation of monocytes with platelets (M+P, solid line) or medium alone (M, dotted line) for different times (n = 1). Dot plots show flow cytometry analysis after 48 h culture. Accumulated results from n = 10 experiments showing percentage of CD14^+^CD16^+^ monocytes (**C**) and MPA (**D**) after co-incubation of monocytes with platelets (M+P) or medium alone (M). (**E**), Regression analysis of MPA level *vs* percentage of CD14^+^CD16^+^ cells at 48 h. (**F**), Percentage of CD14^+^CD16^+^ cells (upper panel) and MPA (lower panel) after co-incubation of monocytes with platelets for different times, either in the presence of isotype antibody (solid line) or anti-PSGL-1 blocking antibody (dotted line) (n = 4). **, *** p<0.01 and <0.001 respectively *vs* anti-PSGL-1.

An initial experiment was performed to study the kinetics of CD16 expression and MPA formation, when monocytes in culture were co-incubated with either autologous platelets or medium alone, for up to 48 h. Monocytes in medium alone showed a progressive time-dependent increase of CD16 cell surface expression. Co-incubation with platelets up-regulated CD16 expression to a greater degree from 18 h onwards (Figure 3A). MPA formation increased in a time-dependent manner, even when monocytes were incubated with medium alone; this reflects an unavoidable degree of platelet contamination of the monocyte sample, with resultant MPA formation. Nevertheless, the addition of exogenous platelets to the culture medium substantially augmented MPA formation, at all time points examined from 18 h onwards (Figure 3B).

On the basis of these results, in subsequent experiments the effect of platelets (or corresponding medium) on CD16 expression in isolated monocytes was investigated at 24 h and 48 h of co-culture, whilst the effect on MPA formation was measured at 48 h. These experiments confirmed that co-incubation with platelets for 48 h caused significant up-regulation of CD16 on monocytes as compared with medium alone (Figure 3C), and an increase in MPA formation (Figure 3D). Regression analysis showed a direct relationship between level of MPA formation and percentage of monocytes expressing CD16 at 48 h (Figure 3E).

**Figure 4:**
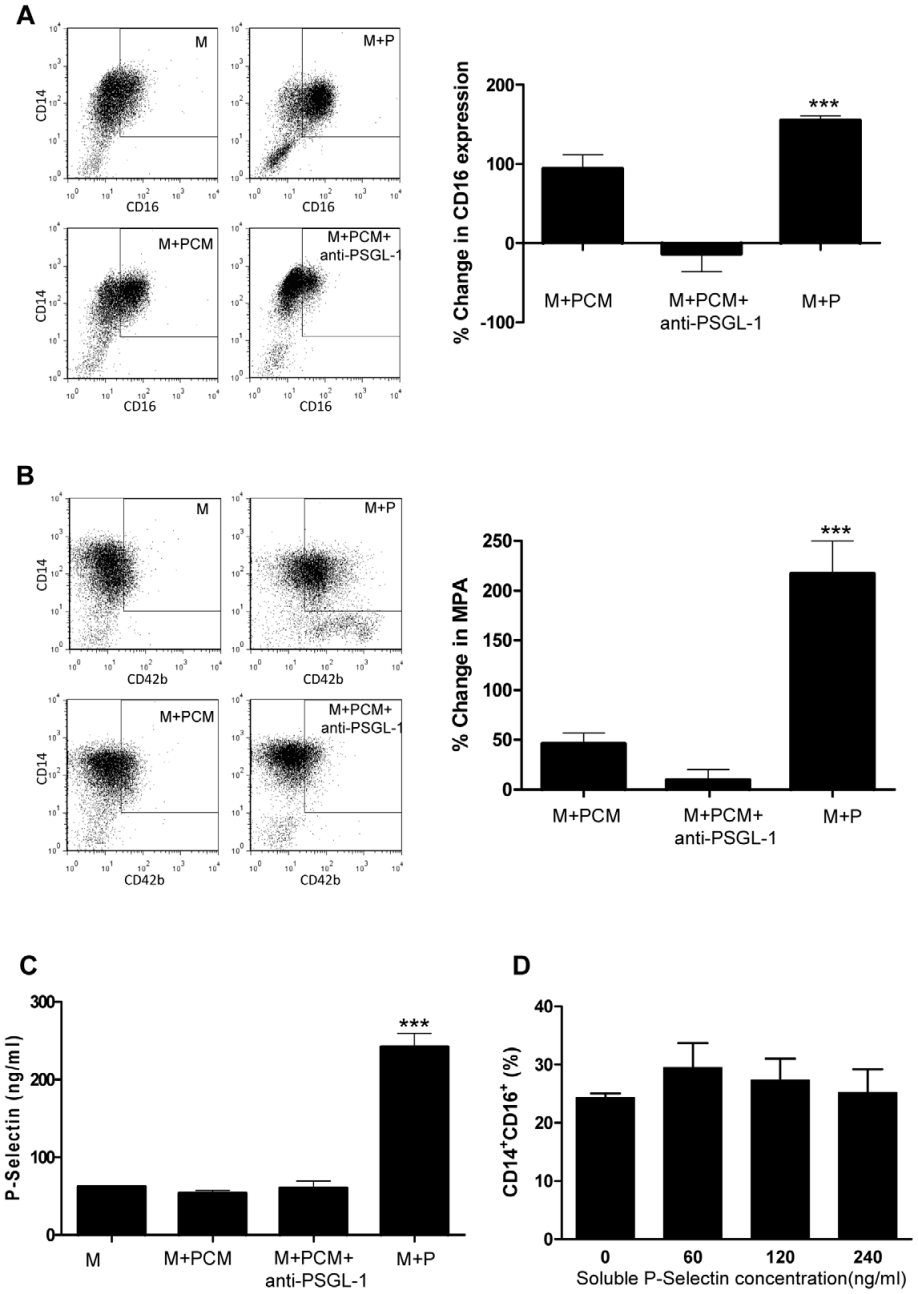
Effect of platelet-free conditioned medium on monocyte phenotype. Percentage of CD14^+^CD16^+^ monocytes (**A**) and MPA (**B**), following 48 h incubation of monocytes in medium alone (M), platelet-free conditioned medium either in the absence (M+PCM) or presence of anti-PSGL-1 blocking antibody (M+PCM+anti-PSGL-1), or platelets (M+P) (n = 3 for each). (**C**), Soluble P-selectin measured in the supernatant of monocytes cultured for 48 h under different experimental conditions as shown. (**D**), Percentage of CD14^+^CD16^+^ monocytes after 48 h exposure to different concentrations of soluble P-selectin. *** p<0.001 vs M.

Although CD16 induction was also seen in monocytes incubated with PCM for 48 h, the percentage of CD16^+^ cells was considerably lower than that achieved by incubation with platelets (Figure 4A). As expected, MPA levels following monocyte culture in PCM was unchanged compared to monocytes in medium alone (Figure 4B), as were levels of soluble P-selectin in the medium (Figure 4C). However, soluble P-selectin levels were much greater following monocyte co-culture with platelets (Figure 4C).

In order to ascertain whether P-selectin binding to monocytic PSGL-1 is in itself sufficient to induce the phenotypic changes observed in monocytes, we examined the effect of co-incubation with anti-human PSGL-1 blocking antibody or isotype control. Blockade of PSGL-1 abrogated MPA formation from monocytes in the presence of platelets, and also markedly reduced the degree of induction of CD16 expression (Figure 3F). Similarly, anti-PSGL-1 blocking antibody abolished the PCM-induced up-regulation of CD16 on monocytes (Figure 4A). However, soluble P-selectin alone, when added to the culture medium of CD14^+^CD16^−^ monocytes, exerted no demonstrable effect on CD16 expression in monocytes, even at concentrations as high as those found in the supernatants of monocyte-platelet co-cultures (Figure 4D).

### CD14^+^CD16^+^ monocytes exhibit increased adhesiveness to activated endothelium

**Figure 5:**
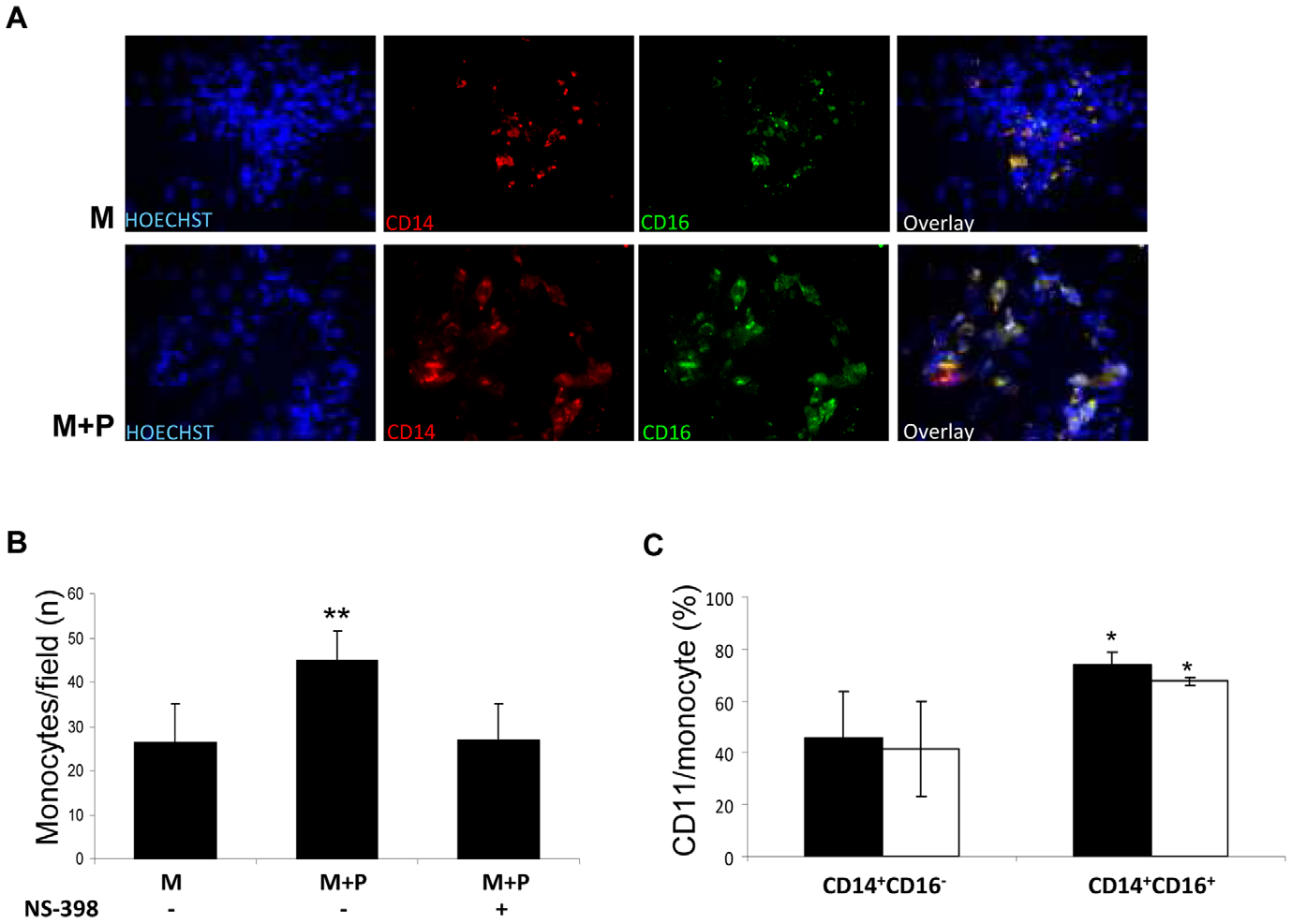
Effect of platelets on monocyte adhesiveness to endothelial cells and expression of adhesion molecules. (**A**), Photomicrographs showing CD14-PE (red) and CD16-FITC (green) staining of monocytes adhering to TNF-α-pre-activated HUVEC (stained with HOECHST, blue), following 48 h culture in medium alone (M) or in the presence of platelets (M+P). (**B**), Number of monocytes/field following 48 h pre-culture in medium alone (M), in the presence of platelets (M+P) either with or without NS-398 (n = 3). ** p<0.01 vs M. (**C**), Expression of CD11b (filled bars) and CD11c (open bars) in the same cellular suspension tested in the HUVEC adhesion assay, in monocyte subpopulations classified according to CD14 and CD16 positivity. *p<0.05 *vs* CD14^+^CD16^−^.

In monocytes co-incubated with platelets as well as in those maintained in medium alone, cells adhering to TNF-α-pre-activated HUVEC were almost exclusively CD14^+^CD16^+^; only occasional monocytes expressing CD14 but not CD16 were observable (Figure 5A). CD14^+^CD16^+^ cells showed a higher expression of both CD11b and CD11c compared with the CD14^+^CD16^−^ subtype (Figure 4C). Pre-treatment of monocytes with platelets increased the number of CD14^+^CD16^+^ monocytes attaching to HUVEC (Figure 5A and 5B).

### COX-2 induction and consequent PGE_2_ generation underlies the phenotypic changes observed in monocytes in response to interaction with platelets

**Figure 6:**
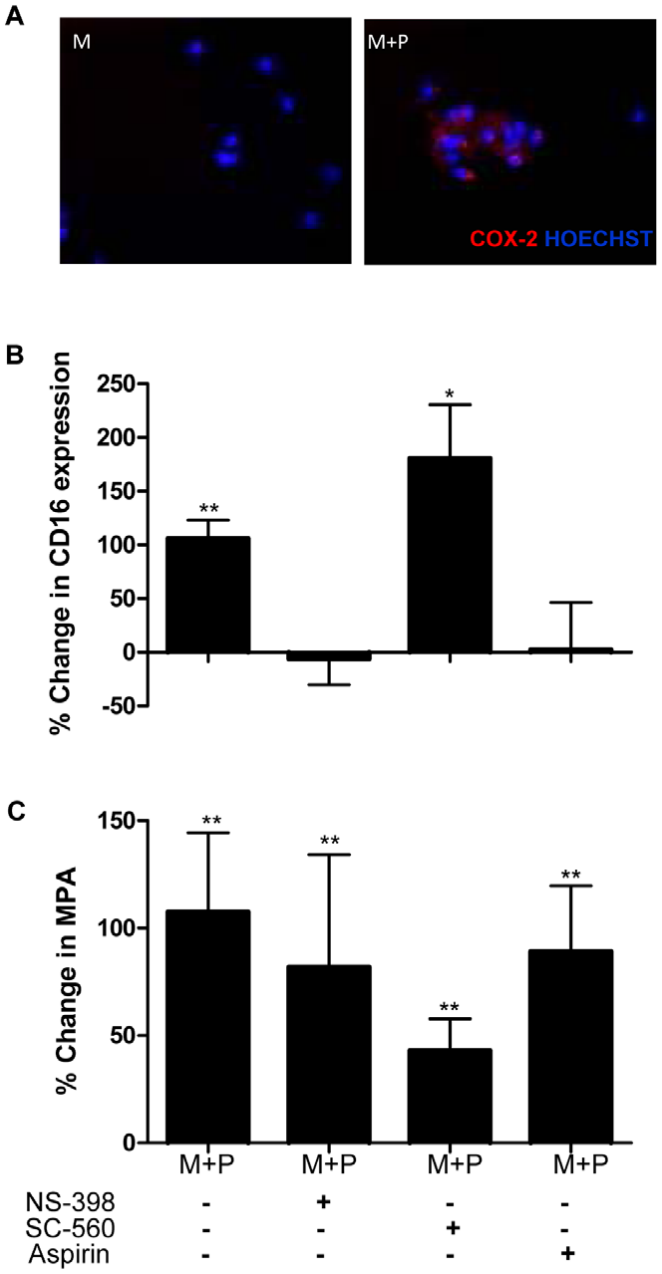
Platelet-dependent COX-2 up-regulation in monocytes and effect of COX inhibitors on monocytic CD16 expression and MPA formation. (**A**), Immunofluorescence staining for COX-2 (red) in monocytes cultured in medium alone (M) or with platelets (M+P) for 18 h. Photomicrographs are representative of n = 3 experiments. Change in CD16 expression (**B**) and MPA (**C**) in monocytes co-cultured with platelets, expressed as % change in CD14^+^CD16^+^ and CD14^+^CD42b^+^ cells respectively, as compared to monocytes cultured in medium alone, after 48 h. Also shown are the effects of NS-398, SC-560 and aspirin addition to monocyte-platelet co-culture (n = 3–4). *, ** p<0.05 and <0.01 respectively *vs* M.

As previously reported [10], co-incubation with platelets for 18 h induced COX-2 expression in monocytes (Figure 6A). Since this effect temporally preceded the phenotypic and functional changes described above in response to monocyte-platelet co-culture (Figure 3), we wished to determine whether COX-2 induction was responsible for inducing these changes, by examining the effect of COX-2 inhibition on monocyte phenotype and function. Platelet-dependent up-regulation of CD16 on monocytes was markedly reduced by the COX-2 selective inhibitor NS-398, while no effect was observed in response to the COX-1 selective inhibitor SC-560; aspirin also reduced platelet-dependent up-regulation of monocytic CD16, to a degree comparable to NS-398 (Figure 6B). MPA formation was not affected by SC-560, NS-398 or aspirin (Figure 6C). Treatment with NS-398 also led to a marked reduction in the number of monocytes adhering to TNF-α-pre-activated HUVEC (Figure 5B) As before, we found that CD14^+^CD16^+^ cells exhibited an advantage in binding to HUVEC over the CD14^+^CD16^−^ subset.

**Figure 7:**
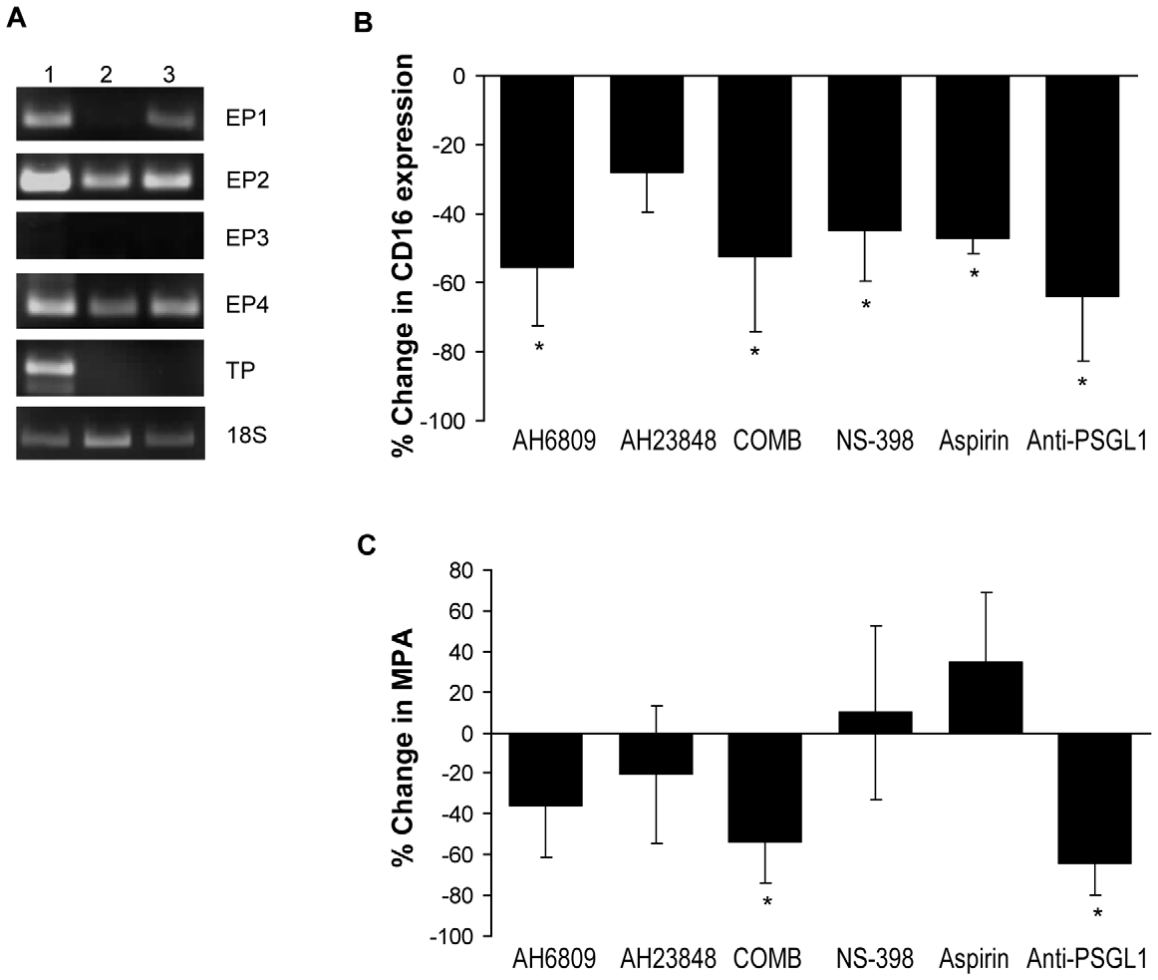
Role of COX-2 up-regulation and of PGE_2_ on platelet-dependent monocytic CD16 expression and MPA formation. (**A**) mRNA level of EP and TP receptors in freshly isolated monocytes (lane 1), and after 48 h culture either with medium alone (lane 2) or platelets (lane 3). 18S ribosomal RNA expression is shown as housekeeping RNA. Experiment is representative of n = 3. Effects of AH6809 and AH3848, alone or in combination (COMB), NS-398, aspirin or anti-PSGL-1 blocking antibody on CD16 expression (**B**) and MPA (**C**) in monocytes co-cultured with platelets for 48 h, expressed as % change in CD14^+^CD16^+^ and CD14^+^CD42b^+^ as compared to monocytes co-cultured with platelets alone. * p<0.05 *vs* M+P.

In order to clarify whether the effects on CD16 expression obtained with NS-398 and aspirin, but not with SC-560, were attributable to differential responsiveness of monocytes to the COX-1- and COX-2-derived prostanoids thromboxane A_2_ and PGE_2_ respectively [15], the mRNA expression of TP and EP receptors was studied (Figure 7A). TP expression was found in CD14^+^CD16^−^ monocytes immediately after isolation, whilst after 48 h incubation, either in medium alone or with platelets, it became undetectable. Freshly isolated CD14^+^CD16^−^ cells expressed PGE_2_ receptors, mainly EP2 and EP4 isoforms, with only small amounts of EP1 and no EP3 mRNA detectable. After 48 h incubation in medium alone, mRNA for EP2 and EP4 were still detectable, albeit at lower levels than in freshly isolated cells; moreover, EP1 mRNA also became undetectable. Notably, when monocytes were co-incubated with platelets for 48 h, the decrease in mRNA for EP1, EP2 and EP4 was less than that observed in monocytes cultured in medium.

We next examined the effect of different PGE_2_ receptor antagonists on platelet-dependent CD16 up-regulation in monocytes (Figure 7B) as well as on MPA formation (Figure 7C). The EP1/EP2-selective antagonist AH6809 markedly reduced monocytic CD16 up-regulation. Although the EP4-selective antagonist AH23848 appeared also to decrease CD16 up-regulation, this did not reach significance, and there was no additive effect of AH23848 and AH6809, suggesting that the EP1 and/or EP2 are predominantly involved in modulating monocytic CD16 up-regulation in response to platelet interaction. Moreover, the effect of AH6809 on the CD16 increase was similar to that seen in response to NS-398, aspirin or anti-PSGL-1 blocking antibody (Figure 6B). The two EP antagonists also reduced formation of MPA in vitro, with this effect being significant when they were used in combination rather than singly; and their combined effect was comparable to that in response to anti-PSGL-1 blocking antibody (Figure 7C).

## Discussion

The present data demonstrate that MPA formation gives rise to a phenotypic change of circulating monocytes toward CD16^+^ cells, which display increased endothelial adhesiveness. We have shown that this change is mediated by platelet-dependent COX-2 up-regulation and consequent PGE_2_ synthesis in monocytes, and that this process can be prevented by selective blockade of either COX-2 enzymatic activity or EP receptors.

CD14^+^CD16^+^ cells identify a subgroup of circulating monocytes with higher pro-inflammatory activity than the “classical” CD14^+^CD16^−^ subpopulation [13]. To date, no direct evidence exists in the literature that double positive cells originate from “classical” monocytes. This study is the first to show that the presence of activated platelets, with consequent MPA formation, induces CD16 up-regulation on CD14^+^CD16^−^ monocytes. Although other groups have reported that platelets can alter monocytic CD16 expression [16], this has been interpreted as an effect on terminal monocytic maturation toward macrophages; however, our *in vitro* results together with our *in vivo* data point to a change in circulating monocyte phenotype rather than a terminal differentiation event. Importantly, due to the isolation techniques used in previous studies (elutriation of peripheral blood mononuclear cells), circulating double positive monocytes were included in the cells under investigation; and since CD16 antigen was measured in the cell supernatant, the prior presence of CD14^+^CD16^+^ monocytes is likely to have affected the results obtained in such studies. Our *in vitro* experiments were performed specifically with isolated CD14^+^CD16^−^ monocytes, using flow cytometry which allowed the direct detection of CD16 on their cellular surface, enabling us to measure the percentage of cells developing positivity for the CD16 marker over time. Moreover, we have quantified the degree of interaction between monocytes and platelets by measuring monocyte-platelet complexes formed *in vitro*. Interaction with platelets up-regulated CD16 antigen on isolated monocytes, with this effect being directly related to the level of MPA. Although simple co-incubation of CD14^+^CD16^−^ monocytes with human recombinant P-selectin did not induce CD16 expression, anti-PSGL-1 blocking antibody, which we have found to abrogate MPA formation,^5^ abolished platelet-induced over-expression of CD16 on monocytes, demonstrating that physical contact of monocytes with platelets is critical in this process, most likely by P-selectin/PSGL-1 binding sustaining multiple receptor-ligand interactions between these cells which trigger the final phenotypic changes.

On the other hand, our experiments suggest that soluble mediators released during the process of platelet activation also exert an important modulatory effect on monocyte phenotype, since CD16 up-regulation was induced by PCM, albeit to a much lower extent than platelets. Nevertheless, PSGL-1 blockade completely abolished both platelet-dependent and PCM-dependent CD16 up-regulation, suggesting that engagement of monocytic PSGL-1 by either soluble or platelet-associated P-selectin is necessary but not sufficient in itself to induce platelet-dependent change in monocyte phenotype.

This study provides important information as regards the mechanism by which platelet-monocyte interaction modulates monocyte phenotype and function, involving platelet-dependent COX-2 up-regulation in monocytes. The selective COX-2 inhibitor NS-398 not only prevented the increased CD16 expression on monocytes co-incubated with platelets, but also reduced monocytic interaction with activated endothelium. In parallel with these findings, selective blockade of EP1/EP2 mirrored the effects observed with COX-2 inhibition. Of note, we found that aspirin counteracted platelet-dependent up-regulation of CD16 on monocytes co-incubated with platelets to a similar extent as NS-398. Others have demonstrated that aspirin, used at concentrations similar to that used in our experiments, modulates PGE_2_ synthesis in monocytes and gives rise to similar effects as those caused by COX-2 specific inhibition [17]; additionally, the COX-1 selective inhibitor SC-560 has been found to be less effective than the COX-2 selective inhibitor NS-398 in reducing PGE_2_ production by bone marrow-derived dendritic cells [18]. It is likely that, in our in vitro experiments, aspirin blocked monocytic PGE_2_ production by COX-2 inhibition, since the same effect on CD16 up-regulation was seen with NS-398 but not with SC-560.

The phenotypic changes induced by MPA formation were found to modulate adhesiveness of monocytes to endothelium. These experiments were performed under static conditions, and in future studies it will be instructive to also assess monocyte adhesion to endothelium under conditions of flow. Nevertheless, our data suggest that CD14^+^CD16^+^ monocytes have greater potential to adhere to activated endothelium than CD14^+^CD16^−^ monocytes. In our adhesion assay, monocyte interaction with endothelial cells occurred through a mechanism independent of bridging via activated platelets. Indeed, the presence of the COX-2 inhibitor in our co-culture experiments reduced monocytic adhesion to endothelial cells, despite the fact that the same level of MPA was present in the cell suspension added. The reduction in CD14^+^CD16^+^ monocytes in response to COX-2 inhibition resulted in reduced binding of isolated monocytes to activated endothelium. The different adhesive properties of the various monocytic subsets may be attributed to differential expression of adhesion molecules on their cellular surface. CD11b and CD11c have been recognized as crucial mediators of monocyte-endothelium interaction [19], [20]. We observed that CD16^+^ cells, both those developed in vitro and those found in the peripheral blood of healthy subjects, display higher levels of these integrins on their surface compared to monocytes positive for CD14 only.

We also observed that other molecules involved in the activation of monocytes and their pro-inflammatory activity [21], [22], namely TLR-2 and TLR-4, are expressed on CD14^+^CD16^+^ monocytes to a higher degree than on other subsets. CD14^+^CD16^+^ cells did not differ from the other monocyte subpopulations in ability to bind activated platelets and form MPA, at least in healthy subjects; accordingly, in our influenza immunization study, similar levels of MPA formed by each subset of monocytes were observed both at baseline and post-immunization. Therefore, the increased levels of MPA seen in the context of an acute inflammatory state, such as that induced by influenza immunization, are likely attributable to increased platelet activation. In our experimental model of inflammation, we found that the increase in hs-CRP, activation of platelets and consequent MPA formation are accompanied by an increase in the percentage of CD16^+^ monocytes in the circulation, mainly driven by expansion of the CD14^high^CD16^+^ subpopulation. It cannot be excluded that the vaccine *per se* drives the phenotypic changes observed on circulating monocytes. However, the level of CD14^high^CD16^+^ cells was found to be strongly and directly related to the percentage of CD62P^+^ platelets, suggesting that the degree of platelet activation influences the phenotype of monocytes in the peripheral blood by shifting them towards positivity for CD16. Although these results suggest that accumulation of CD14^high^CD16^+^ cells *in vivo* is a platelet-driven phenomenon, this would require confirmation in future *in vivo* studies, for example using animal models where platelets are depleted. In future studies, it would also be useful to ascertain if the effects of COX inhibition which we have here reported *in vitro* can be replicated *in vivo* in human subjects.

Our data provide evidence that a host of modifications occur in the phenotype of circulating monocytes in response to a pro-inflammatory stimulus, rendering them more pro-inflammatory and with increased endothelial adhesiveness, and that these are strongly related to – and are likely to be driven by – the extent of platelet activation. Our findings shed new light on the relevance of platelet-monocyte interactions in the pathophysiology of inflammation, and may provide a novel therapeutic target in patients with inflammatory disorders.

## Materials and Methods

### Ethics statement

The study was approved by St Mary's Hospital Research Ethics Committee, London, UK. All participants gave written informed consent.

### Effect of influenza immunization on circulating MPA and monocyte phenotype

Twelve healthy subjects (9 male, 3 female; age 25–30 years) were recruited sequentially, from employees of Guy's and St Thomas' NHS Foundation Trust who were attending the Trust's Occupational Health service to receive influenza immunization. Subjects were studied immediately before, and 2 days following, influenza vaccine administration. At the first visit, blood (32 ml) was taken for full blood count and biochemistry screening, as well as for high sensitivity C-reactive protein (hs-CRP) assay, measurement of CD62P+ platelets (reflecting degree of platelet activation), determination of MPA level and characterization of monocytes. At the second visit, blood (4 ml) was once again taken, for repeat determination of hs-CRP, CD62P+ platelets and MPA, as well as monocyte characterization.

Percentage of CD62P+ platelets was analyzed by flow cytometry (FACSCalibur, Becton Dickinson (BD), Oxford, UK) on whole blood immunostained with fluorescein isothiocyanate (FITC)-conjugated anti-human CD42b and allophycocyanin (APC)-conjugated anti-human CD62P (BD Bioscience, UK). 10,000 events in total were acquired within the platelet gate, as identified on forward and side light scatter plot. MPA determination and monocyte characterization were performed by whole blood flow cytometry, as described below.

### Monocyte characterization and MPA measurement in peripheral blood

In all further experiments, a separate cohort of 15 healthy subjects (10 male, 5 female; age 25–35 years) was studied. Monocyte characterization and MPA measurement were performed by flow cytometry analysis on whole blood (4 ml) collected in sodium citrate (0.3% final concentration). Immediately after venepuncture, 100 µl blood was immunostained with different combinations of the following antibodies: peridinin chlorophyll protein complex (PerCP)-conjugated anti-human CD14, FITC-conjugated anti-human CD16, APC-conjugated anti-human CD42b or CD62P, phycoerythrin (PE)-conjugated anti-human CD11b or CD11c (all from BD Bioscience), and APC-conjugated anti-human Toll-like receptor (TLR)-2 or 4 (eBioscience, UK). Isotype control antibodies were used as negative control. After red cell lysis using FACS lysing solution (BD Bioscience), samples were fixed in 1% paraformaldehyde and kept at 4°C until analyzed within a maximum of 48 h from sample preparation.

Forward and side light scatter parameters were used to access the monocyte population, and a total of 20,000 events acquired. The negative and positive delineators were determined from the isotype control fluorescence.

Monocyte subsets were identified by double immunostaining for CD14 and CD16. The percentage of CD14+ cells also expressing CD42b (all platelets) or CD62P (activated platelets) was also calculated and taken as representative of MPA. By gating for each monocyte subtype, percentage of cells positive for CD11b/c or TLR-2/4 as well as the mean fluorescence intensity (MFI) for CD11b/c or TLR-2/4 was analyzed. Post-acquisition analysis was performed using FlowJo software (Tree Star, Ashland, OR).

### Monocyte and platelet co-culture

Whole blood (30 ml) collected in EDTA was subjected to Lymphoprep gradient centrifugation. Mononuclear cells underwent immunomagnetic negative selection for CD14+CD16- monocytes (kit from Invitrogen) according to the manufacturer's instructions. Purity of isolation, as determined by antigen expression (CD14^+^, CD16^−^, CD3^−^, CD4^−^, CD8^−^, CD20^−^) using flow cytometry, was 78–82% in all experiments. Isolated cells were resuspended in serum-free RPMI-1640 (10^6^cells/ml), and transferred to polystyrene tubes.

Platelets were pelleted by centrifugation of plasma at (1400×g, 4°C, 6 min). The pellet was resuspended, under sterile condition, in PBS containing 5 mmol/l EDTA, and platelets were centrifuged (500×g, room temperature, 5 min) and resuspended (25×10^9^ platelets/ml) in serum-free RPMI-1640. Platelet-conditioned medium (PCM) was obtained by stirring isolated platelets in serum-free RPMI-1640 (1200 rpm, 10 min, 4°C), followed by removal of platelets by centrifugation (10,000×g, 3 min, room temperature) and collection of supernatant.

Aliquots of purified autologous platelets or equal volumes of PCM were added to monocyte suspensions (1 ml final volume, monocyte∶platelet ratio 1∶100 in experiments where platelets were added) and incubated at 37°C, 4% CO_2_ for 48 h. Although these experiments were performed in the absence of pharmacological platelet agonists, the platelet isolation and culture procedure prior to addition to monocytes induced platelet activation, with P-selectin expression measured at 87±3%. Co-incubation of monocytes with platelets or PCM was also performed in the presence of monoclonal anti-human PSGL-1 blocking antibody (10 µg/ml; Chemicon), and the effect compared to that obtained with isotype control (mouse anti-human IgG, 10 µg/ml; Chemicon).

Viability of monocytes, as assessed by Trypan Blue dye exclusion, was >95% immediately after their isolation and after 48 h culture, either in the presence or absence of platelets/PCM. CD16 expression and MPA formation were assessed by flow cytometry at different time points. The supernatants were also collected and soluble P-selectin measured according to the manufacturer's instructions (R&D System, UK).

In separate experiments, monocytes were treated for 48 h with increasing concentrations of soluble human recombinant P-selectin (0–240 ng/ml, R&D System), and monocytes characterized as before.

### Investigation of the role of COX-2 and prostaglandin E2 in modulating MPA and monocyte phenotype

We investigated cyclooxygenase type 2 (COX-2) induction in monocytes co-incubated for 18 h with either platelets or medium alone. Following such incubation, cells were permeabilized with Triton X-100 0.01%, and immunostained for 20 min at 4°C in PBS/0.2% bovine albumin serum containing PE-conjugated mouse anti-human COX-2 antibody (Calbiochem,UK; 1 µg/ml). After washing in PBS and fixation in 1% paraformaldeyde, cells were resuspended in 10 µl PBS, seeded on a microscope slide and examined under fluorescence microscopy (40× magnification, Zeiss LSM, 510 META).

CD16 expression and MPA formation were analyzed following monocyte co-incubation with platelets for 48 h, either in the presence or absence of different COX inhibitors: NS-398 (10 µM, a COX-2-selective inhibitor) [23], SC-560 (30 nM, a COX-1-selective inhibitor) [23] and aspirin (0.5 mM, a non-selective COX inhibitor). The effect of prostaglandin E_2_ (PGE_2_) receptor blockade on CD16 expression and MPA level was also examined using the EP1/EP2-selective antagonist AH-6809 (10 µM; Cayman) and the EP4-selective antagonist AH-23848 (10 µM; Cayman) [24], either alone or in combination. NS-398, aspirin and EP antagonists were resuspended in DMSO/PBS solution (1∶3), whilst SC-560 was resuspended in ethanol. In all experiments, the effects of each antagonist were compared to those obtained using the respective vehicle.

Expression at the mRNA level of the thromboxane receptor (TP) (α-isoform) and the different PGE_2_ receptors (EP1, EP2, EP3 and EP4) was studied by semi-quantitative reverse transcriptase polymerase chain reaction (RT-PCR), in freshly isolated monocytes and those cultured for 48 h either in medium alone or with platelets. Total RNA was extracted by phenol-chloroform extraction (TRIZOL, Invitrogen) and resuspended in RNase- and DNase-free water. RNA concentration was analyzed by NanoDrop and 500 ng total RNA used for cDNA synthesis. Equal aliquots of cDNA were then PCR-amplified by Taq Polymerase (Invitrogen, UK) in a 40 µl reaction containing 1× Buffer, 250 µM dNTPs, 4 mM MgCl2, 1.25 µM of each primer, 2U Taq. The conditions of amplification were as follows: an initial 5 min at 95°C for denaturation; 36 cycles of 95°C for 1 min, 60°C for 1 min and 72°C for 1 min 30 s; and a final extension step of 72°C for 10 min. The sense and antisense primers used, and the expected product sizes, are listed in Table 1.

**Table 1:** Primer sequences used for RT-PCR.

GENE	Forward Primer (5′-3′)	Reverse Primer (5′-3′)	Product size (bp)
EP1	TTGTCGGTATCATGGTGGTG	ATGTACACCCAAGGGTCCAG	160
EP2	GTCTGCTCCTTGCCTTTCAC	CGACAACAGAGGACTGAACG	176
EP3	ATCTCAGTCCAGTGCCCAGT	TTTCTGCTTCTCCGTGTGTG	172
EP4	CTGGTGGTGCTGATCTGCT	TATCCAGGGGTCTAGGATGG	150
TP	AGGTGGAGATGATGGCTCAG	CGGCGGAACAGGATATACAC	220

### Monocyte adhesion to endothelial cells

Human umbilical vein endothelial cells (HUVEC) were isolated from fresh umbilical cords obtained from uncomplicated deliveries following healthy pregnancies; these were obtained from the labour ward at St Thomas' Hospital, with written informed consent from the mothers. HUVEC were isolated using previously described methods [25]. When confluent at passage three, HUVEC were seeded onto coverslips in 12-well plates. The following day, HUVEC were stimulated with tumor necrosis factor-α (TNF-α, 10 ng/ml, Invitrogen) for 3 h at 37°C, and then washed with PBS. A cell suspension containing monocytes pre-cultured either in medium alone or with platelets for 48 h was added to the coverslip cultures. After further 2 h incubation at 37°C, non-attached cells were removed by vigorous pipetting. Immunostaining using PE-conjugated anti-CD14 and FITC-conjugated anti-CD16 was then performed at 4°C for 20 min. After washing in PBS twice, HOECHST solution was added for nuclear staining and removed after 2 min incubation, following which cells were fixed in 1% paraformaldeyde. The coverslips were mounted on slides with mounting medium and analyzed by fluorescence microscopy. Cells positive for monocytic markers were counted in ten fields (20× magnification).

### Statistical analysis

All data are presented as mean ± SD. Statistical analysis was performed using GraphPad Prism 4 software. Differences in level of hs-CRP, CD62P+ platelets, MPA and monocyte phenotype before and after influenza vaccine were evaluated by paired Student's *t* test, and the associations between these different parameters were analyzed by least squares and multiple regression analyses. All other statistical comparisons were by ANOVA, with or without repeated measures as appropriate. In all cases, P<0.05 (two-tailed) was taken to indicate statistical significance.
